# Effects of functional variants of vitamin C transporter genes on apolipoprotein E E4-associated risk of cognitive decline: The Nakajima study

**DOI:** 10.1371/journal.pone.0259663

**Published:** 2021-11-15

**Authors:** Koji Hayashi, Moeko Noguchi-Shinohara, Takehiro Sato, Kazuyoshi Hosomichi, Takayuki Kannon, Chiemi Abe, Chiaki Domoto, Sohshi Yuki-Nozaki, Ayaka Mori, Mai Horimoto, Masami Yokogawa, Kenji Sakai, Kazuo Iwasa, Kiyonobu Komai, Mai Ishimiya, Hiroyuki Nakamura, Natsuko Ishida, Yukio Suga, Junko Ishizaki, Akihito Ishigami, Atsushi Tajima, Masahito Yamada

**Affiliations:** 1 Department of Neurology and Neurobiology of Aging, Kanazawa University Graduate School of Medical Sciences, Kanazawa, Japan; 2 Department of Bioinformatics and Genomics, Graduate School of Medical Sciences, Kanazawa University, Kanazawa, Japan; 3 Department of Preemptive Medicine for Dementia, Kanazawa University Graduate School of Medical Sciences, Kanazawa University, Kanazawa, Japan; 4 Department of Physical Therapy, Division of Health Sciences, Kanazawa University Graduate School of Medical Sciences, Kanazawa, Japan; 5 Ishikawa Prefectural Nursing University, Kahoku, Japan; 6 Department of Neurology, Iou Hospital National Hospital Organization, Kanazawa, Japan; 7 Department of Oral and Maxillofacial Surgery, Kanazawa University Graduate School of Medical Sciences, Kanazawa, Japan; 8 Department of Oral and Maxillofacial Surgery, Ryukyu University Hospital, Nishihara, Japan; 9 Clinical Pharmacy and Healthcare Sciences, Faculty of Pharmacy, Institute of Medical, Pharmaceutical & Health Science, Kanazawa University, Kanazawa, Japan; 10 Molecular Regulation of Aging, Tokyo Metropolitan Institute of Gerontology, Tokyo, Japan; Nathan S Kline Institute, UNITED STATES

## Abstract

Apolipoprotein E E4 (APOE4) is a risk factor for cognitive decline. A high blood vitamin C (VC) level reduces APOE4-associated risk of developing cognitive decline in women. In the present study, we aimed to examine the effects of functional variants of VC transporter genes expressed in the brain (*SLC2A1*, *SLC2A3*, and *SLC23A2*) on APOE4-associated risk of developing cognitive decline. This case–control study involved 393 Japanese subjects: 252 cognitively normal and 141 cognitively impaired individuals (87 mild cognitive impairment and 54 dementia). Database searches revealed that rs1279683 of *SLC23A2*, and rs710218 and rs841851 of *SLC2A1* are functional variants that are significantly associated with the altered expression of the respective genes and genotyped as three single nucleotide variants (SNVs). When stratified by SNV genotype, we found a significant association between APOE4 and cognitive decline in minor allele carriers of rs1279683 (odds ratio [OR] 2.02, 95% CI, 1.05–3.87, *p* = 0.035) but not in the homozygote carriers of the major allele. Significant associations between APOE4 and cognitive decline were also observed in participants with major allele homozygotes of rs710218 (OR 2.35, 95% CI, 1.05–5.23, *p* = 0.037) and rs841851 (OR 3.2, 95% CI, 1.58–6.46, *p* = 0.0012), but not in minor allele carriers of the respective SNVs. In contrast, the three functional SNVs showed no significant effect on cognitive decline. Our results imply that functional SNVs of VC transporter genes can affect APOE4-associated risk of developing cognitive decline via altered VC levels in the brain.

## Introduction

Cognitive decline in patients with mild cognitive impairment (MCI) and dementia is a complex trait resulting from the interaction between genetic and environmental factors. The E4 variant of apolipoprotein E (APOE4) is a well-known strong genetic risk factor for Alzheimer’s disease (AD) [1] and confers susceptibility to MCI [2]. In addition, genetic association studies performed using genome-wide association analysis and meta-analysis have shown that *APOE ε4* (*APOε4*) confers susceptibility to vascular dementia and dementia with Lewy bodies [3–7]. APOE4 risk of cognitive decline may be modified by interaction with other factors; however, the role of risk modifiers or interacting factors is not well understood. In our previous longitudinal study, we found that high serum vitamin C (VC) levels during normal cognition reduce APOE4-associated risk of cognitive decline, especially in women [8].

VC is a well-known antioxidant in humans and other mammals [9]. VC is also required for diverse biological processes and is involved in several enzymatic reactions, including the synthesis of collagen and neurotransmitters as a cofactor [10]. Studies in animal models have revealed that VC is maintained at relatively high concentrations in the brain during systematic VC deficiency, suggesting the vital role of VC in the brain [11, 12]. Animal studies have showed that chronic VC deficiency causes cognitive dysfunction via mitochondrial dysfunction and increased oxidative stress [13, 14]. Acute administration of VC improved cognitive function in mouse models of AD and aging [15, 16].

As humans cannot biosynthesize VC, its intake through diet is essential for the maintenance of the normal VC levels. The ingested VC is transported from the digestive tract to the body. VC includes ascorbic acid (ASC; reduced form) and dehydroascorbic acid (DHA; oxidized form). ASC absorption occurs in the body via sodium-dependent VC transporter (SVCT), whereas that of DHA occurs via glucose transporters (GLUTs) [17]. A study using gene-knockout mice showed that SVCT1 (encoded by *SLC23A1*) plays an important role in intestinal absorption and renal reabsorption of ASC [18]. Additionally, the ASC level in neonatal mice with homozygous knockout of SVCT2 (encoded by *SLC23A2*) was undetectable or considerably reduced in various tissues including the brain, compared with that in heterozygous knockout or wild-type pups. Besides, compared with age-matched wild-type mice, newborn and adult mice with heterozygous knockout of SVCT2 have reduced ASC level in the brain and several other tissues [19, 20]. SVCTs and GLUTs transport VC to various organs. The transport of ASC into the brain is mediated by SVCT2 (encoded by *SLC23A2*) [21], whereas DHA uptake in the brain occurs via GLUT1 (encoded by *SLC2A1*) and GLUT3 (encoded by *SLC2A3*) [22, 23]. Depending on the ASC and DHA levels, the intracellular DHA-to-ASC recycling system is activated, and the amount of VC influx and efflux is regulated by SVCT2 and GLUT1/3 [17]. Thus, VC transporters are important in maintaining VC levels in the body, and in particular, SVCT2 and GLUT1/3 are thought to play important roles in the regulation of VC levels in the brain.

To investigate the relationship between cognitive decline and brain VC level in humans, we need to focus on the roles of brain-expressed VC transporters (*SLC23A2*, *SLC2A1*, and *SLC2A3*) in human cognitive function. We hypothesized that genetic variants of VC transporters (*SLC23A2*, *SLC2A1*, and *SLC2A3*) expressed in the brain could affect the risk of developing APOE4-associated cognitive decline. To test this hypothesis, we first selected functional variants of VC transporter genes from database searches, and then performed case–control association analysis to examine the effects of functional variants on the risk of developing APOE4-associated cognitive decline. In addition, we examined the relationship between functional variants of VC transporter genes and cognitive decline.

## Subjects and methods

### Study design and grouping by cognitive function

The Nakajima study is a population-based longitudinal cohort study that investigated cognitive decline in aged Japanese individuals. The study started in 2006 in Nakajima, Nanao District, Ishikawa Prefecture, Japan. Nanao City supported our study and provided resident information. The study included questionnaire about personal lifestyle including medical, educational, smoking, and alcohol history. As a part of the Nakajima study, this study involved individuals aged 65 years or older with normal cognitive function during the 2006–2008 baseline survey. The study design has been described in detail previously [8, 24–26]. We assessed participants’ cognitive status using the Mini-Mental State Examination (MMSE) [27] and Clinical Dementia Rating [28–30]. Diagnosis of dementia was based on the guidelines of Diagnostic and Statistical Manual of Mental Disorders, Third Edition, Revised (DSM-III-R) [31]. MCI was diagnosed based on the general criteria for MCI by the International Working Group [32]. The diagnosis has been described in detail in a previous report [8]. The blood was collected with the consent of the participants, and serum VC level and APOE phenotype determination, and DNA extraction from blood were performed at SRL, Inc. (Tokyo, Japan).

Participants who cooperated with the baseline survey were followed-up, and their cognitive status was evaluated between 2014 and 2017. In the follow-up survey, we assigned participants with normal cognitive function to the normal group and participants with MCI or dementia to the cognitive decline group.

This study was approved by the medical ethics review board of Kanazawa University (Kanazawa, Japan) (approval numbers 257, 698, 721, 933, 1117, 1188, and 2186). All participants provided written informed consent by signing a form that described the purpose and procedures of the study, the voluntary nature of participation, the right to withdraw from the research without prejudice or penalty, and a guarantee of confidentiality and security of personal data.

### Selection and genotyping of the SLC23A2, SLC2A1, and SLC2A3 variants

We hypothesized that genetic variants that alter the function of VC transporters in the brain affect the VC level and APOE4-associated risk of developing cognitive decline. To test this hypothesis, the identification of variants that could affect the function of transporter genes was necessary. Among the three genes, we assumed the following three genetic factors affecting the function of VC transport: gene-overlapping large-scale structural variations (SVs), SNVs and small insertions/deletions (indels) that cause changes in the protein-coding sequences (e.g., frameshift, nonsense, missense, and splice-altering variants), or SNVs and small indels that alter transcript abundance.

Because of the relatively small number of participants in this cohort, we set minor allele frequency (MAF) of 0.15 to detect functional variants with considerable effect on cognitive decline. Using “Genetic Power Calculator” (https://zzz.bwh.harvard.edu/gpc/), this study presented 82.9% power to detect common variants (MAF ≥ 15%) with genotype relative risk of 1.5, assuming 0.05 of type I error rate and 25% of MCI + dementia prevalence [33].

For screening large-scale SVs, we used the Database of Genomic Variants (DGV; http://dgv.tcag.ca/dgv/app/home). In the DGV, we searched for “DGV Gold Standard Variants”, a curated SV, which overlaps with *SLC23A2*, *SLC2A1*, and *SLC2A3* with at least 15% frequency.

For screening SNVs and small indels that alter respective protein sequences of the three genes, we used the “Genome Variation” database at Japanese Multi Omics Reference Panel (jMorp; https://jmorp.megabank.tohoku.ac.jp/202001/) that provided allele frequencies for SNVs or indels with MAF ≥ 0.01% from the whole-genome sequencing analysis of approximately 8300 Japanese individuals. We selected the following variants with MAF ≥ 15%: frameshift, missense, and splice donor/acceptor variants in *SLC23A2*, *SLC2A1*, and *SLC2A3*, respectively.

For screening SNVs and small indels that alter the amount of transcription, we first obtained all variants with MAFs ≥ 15% in *SLC23A2*, *SLC2A1*, and *SLC2A3* from the “Genome Variation” database at jMorp. Next, using the Regulome database (http://regulomedb.org/), we searched for variants with score 1 (score 1a – 1f) having evidence of expression quantitative trait loci (eQTLs) and transcriptional factor binding or DNase peak among the obtained variants with MAFs ≥ 15%. We then used the “GTEx” Portal database (https://gtexportal.org/home/) to determine whether the “Regulome DB score 1” variants were eQTLs, which could affect the expression of *SLC23A2*, *SLC2A1*, and *SLC2A3*. If multiple functional variants were found in a single gene from database searches, pairwise linkage disequilibrium values (*r*^2^-values) between the variants were evaluated using the LDlink (https://ldlink.nci.nih.gov/) and JPT (Japanese in Tokyo, Japan) databases.

### Genotyping of the SLC23A2, SLC2A1, and SLC2A3 variants

Genotyping of functional variants in all the participants was performed by TaqMan SNP Genotyping Assays (Thermo Fisher Scientific, Waltham, MA, USA) using 7500 Fast Real-Time PCR System (Thermo Fisher Scientific). Each PCR mixture contained 4 ng of each DNA, 5 μL of TaqMan Genotyping Master Mix (Thermo Fisher Scientific), and 0.5 μL of TaqMan Genotyping Assay Mix (Thermo Fisher Scientific).

### Statistical analyses

Chi-square test for categorical variables and Student’s *t*-test for continuous variables were performed to compare the characteristics of participants at baseline and follow-up survey between the normal cognition and cognitive decline (MCI or dementia) groups. To assess APOE4-associated risk of developing cognitive decline, a multivariate logistic regression analysis was performed using the significant variables (*p* < 0.1) obtained from the univariate analysis, except for MMSE at the follow-up survey. Because of sex differences in blood VC levels [34], VC values standardized by sex were used in the multivariate analysis. Variables that were statistically significant in the multivariate analysis were used as covariates in the subsequent analyses.

The *p* values in the Hardy–Weinberg equilibrium test were calculated based on the genotypic distribution of functional variants. To assess the effects of VC transporter genes on APOE4-associated risk of developing cognitive decline, participants were stratified based on the genotype of each variant into two strata: homozygote carriers of the major allele and carriers of the minor allele. In the stratified analysis by variant genotype, a multivariate logistic regression analysis was performed to examine whether there was a significant association between the APOE4 phenotype and cognitive decline (MCI or dementia) with adjustments for significant covariates. The *p* value, odds ratios (ORs), and 95% confidence interval (CI) of APOE4-associated risk of developing cognitive decline (MCI or dementia) were calculated for each stratum. Assuming a type I error rate of 0.05, statistical power to detect APOE4-associated risk of developing cognitive decline (MCI or dementia) in each stratified analysis was calculated based on the effect size of APOE4 and the frequency of APOE4 phenotype in the follow-up survey of this study. A multivariate logistic regression analysis was also performed to assess genetic association between functional variants of VC transporter genes and cognitive decline (MCI or dementia) in the following two genetic models: dominant model (minor allele) and log-additive model. The *p* value, OR, and 95% CI of the functional variants for cognitive decline (MCI or dementia) were calculated for each genetic model.

Statistical analyses were performed using R statistical environment (https://www.r-project.org/). The significance level was set at 5%.

## Results

**Fig 1:**
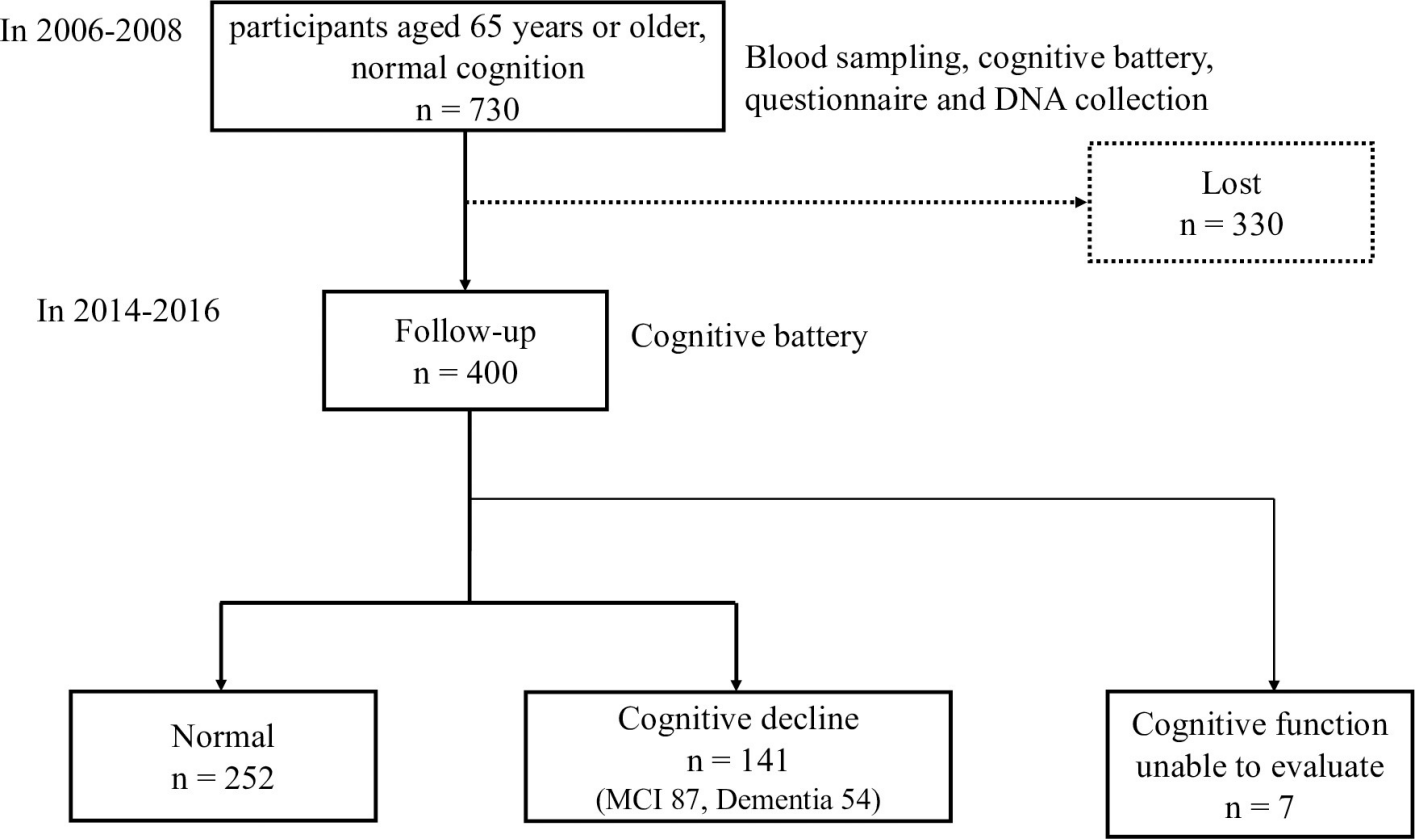
Design of the case–controlled study. Baseline survey was conducted between 2006 and 2008. The participants aged 65 years or older with normal cognition were selected. In the baseline survey, participants took cognitive battery, underwent blood sampling, and responded to a questionnaire, including medical history. DNA was extracted from the blood. Between 2014 and 2016, follow-up survey was conducted, and the cognitive status was evaluated again. Based on the cognitive status, the participants were classified into normal group (normal cognition) or cognitive decline group (MCI or dementia).

In the baseline survey, 923 individuals aged 65 years or older were included in the study. Among the 923 participants, by applying the previously mentioned diagnostic criteria, 730 were identified with normal cognitive function in the baseline study. In the follow-up survey, 400 participants were included, among which 252 were classified into the normal group and 141 in the cognitive decline group (MCI: 87 and dementia: 54), except for seven participants whose cognitive function could not be determined (Fig 1). The details of the characteristics of participants at baseline and follow-up surveys are summarized in Table 1. The participants in the cognitive decline group in follow-up survey were older than those in the baseline survey, and had a longer follow-up period, shorter education period, and lower MMSE score in both baseline and follow-up surveys, and a higher frequency of APOE4 phenotype than the normal cognition group in the follow-up survey. In female participants of the cognitive decline group, the blood VC level was significantly lower in the baseline survey than that in the follow-up survey. The multivariate logistic regression analysis adjusted for the effects of age, follow-up period, education period, MMSE points at baseline, frequencies of hypertension and hyperlipidemia, standardized VC levels, and sex showed that APOE4 is an independent risk factor for cognitive decline (MCI or dementia) (S1 Table: *p* = 0.027, OR = 1.91, and 95% CI, 1.10–3.33). In addition, the lost to follow-up participants (n = 330) were older, had lower MMSE scores at baseline, and had a shorter education period than the follow-up participants (n = 400) (S2 Table).

**Table 1:** Characteristics of the participants with normal cognition (n = 393) at baseline (2006–2008) who presented with normal cognition (n = 252) or MCI/dementia (n = 141) in the follow-up survey (2014–2016).

		Normal cognition at follow-up survey	MCI or dementia at follow-up survey	*p* value
N (Male/Female)		252 (77/175)	141 (53/88)	0.18
Baseline age		71.4 ± 4.6	74.6 ± 5.6	5.91 × 10^−8^ *
Follow-up period (yrs)		8.12 ± 1.00	8.36 ± 1.09	0.030 *
Education period (yrs)		9.97 ± 2.33	9.18 ± 2.21	5.83 × 10^−4^ *
Baseline Vitamin C (μg/mL)				
	Male	5.00 ± 3.03	5.39 ± 3.16	0.47
	Female	8.49 ± 3.27	7.25 ± 3.28	4.01 × 10^−3^ *
Baseline MMSE (points)		27.8 ± 1.9	26.7 ± 2.2	1.41 × 10^−6^ *
Follow-up MMSE (points)		28.0 ± 1.8	22.8 ± 5.4	6.52 × 10^−21^ *
APOE E4 positive, N(%)		43 (17.0%)	40 (29.0%)	0.014 *
Hypertension, N(%)		154 (61.1%)	73 (51.7%)	0.083
Hyperlipidemia, N(%)		94 (37.3%)	40 (28.4%)	0.095
Diabetes mellitus, N(%)		39 (15.5%)	23 (16.3%)	0.89
Alcohol, N(%)		96 (38.1%)	64 (45.3%)	0.34
Smoking, N(%)		56 (22.2%)	39 (27.6%)	0.39

Statistical analysis was performed by chi-square test and Student’s *t*-test between the groups.

* *p* < 0.05.

In the case of SVs overlapped with *SLC23A2*, *SLC2A1*, and *SLC2A3* in DGV, we found 1 “DGV Gold Standard” SV in *SLC23A2*, 0 SV in *SLC2A13*, and 7 SVs in *SLC2A3*, all of which did not meet the criterion of at least 15% frequency. In the search with the “Genome Variation” database at jMorp, we found no SNV and small indels altering the protein sequences of *SLC23A2*, *SLC2A1*, and *SLC2A3*, with MAF ≥ 15% in the Japanese population. Therefore, we focused on SNVs and small indels altering the transcript abundance of *SLC23A2*, *SLC2A1*, and *SLC2A3* in the subsequent analyses.

**Fig 2:**
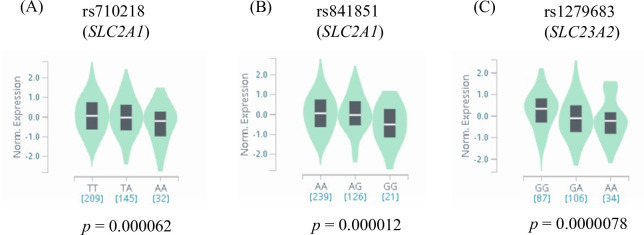
Genotype-specific differences in transcript levels of vitamin C transporter gene. These data were cited from the GTEx Portal on 11/01/2020 (https://gtexportal.org/). In the “GTEx” Portal database, a significant correlation was observed between the genotype of each SNV and the transcript level of *SLC23A2* or *SLC2A1* in human tissues. The *p* value indicates a significant difference between gene expressions by genotype. The genotype-expression association data for tissues with the lowest *p* values are presented for the 49 human tissues in the “GTEx” Portal database. (A) The transcription level of *SLC2A1* in the left ventricle by rs710218 genotype. (B) The transcription level of *SLC2A1* in the left ventricle by rs841851 genotype. (C) The transcription level of *SLC23A2* in the spleen by rs1279683 genotype.

Among 226 SNVs and small indels with MAF ≥ 15% in *SLC23A2* from the “Genome Variation” database at jMorp, only one SNV (rs1279683) was classified as a functional variant with the “Regulome DB” score 1d, which had evidence of eQTLs and functional genomic regions such as transcriptional factor binding sites. Using the “GTEx” Portal database, rs1279683 was proven to be an eQTL of *SLC23A2* in the spleen tissue, where the most significant association between the genotype of rs1279683 and the *SLC23A2* transcription level was observed among 49 human tissues in the “GTEx” Portal database (Fig 2). We also found that the transcription level of *SLC23A2* in several brain tissues was significantly different according to the genotype of rs1279683 (data not shown). In *SLC2A1*, we found two SNVs (rs710218 and rs841851) as functional variants with the “Regulome DB” score 1b among 86 SNVs and small indels with MAF ≥ 15% in the “Genome Variation” database at jMorp. The two SNVs were not in high linkage disequilibrium with each other (pairwise *r*^2^ = 0.48) in the JPT database. The “GTEx” Portal database showed that rs710218 and rs841851 were eQTLs of *SLC2A1* in the left ventricle tissue, with the lowest *p* value for the association between the genotype of each SNV and the *SLC2A1* transcript level among the 49 human tissues (Fig 2). In the brain tissues, we confirmed that the transcription level of *SLC2A1* in the frontal cortex and the nucleus accumbens tended to differ depending on the rs841851 and rs710218 genotypes, respectively, in the “GTEx” Portal database (data not shown). As for *SLC2A3*, we found six SNVs (rs4883461, rs933552, rs7975829, rs12313154, rs7309332, and rs11610602) as functional variants with the “Regulome DB” score 1f among 99 SNVs and small indels with MAF ≥ 15% in the “Genome Variation” database at jMorp. However, there was no evidence showing that the six SNVs were eQTLs of *SLC2A3* in any tissues in the “GTEx” Portal database.

The database searches revealed rs1279683 (*SLC23A2*), rs841851 (*SLC2A1*), and rs710218 (*SLC2A1*) as functional variants of VC transporters in the brain. These three SNVs were genotyped in 388 participants, excluding five participants in the normal group who had poor DNA quality. The genotype frequencies of the three SNVs are listed in S3 Table. No deviation was found in the genotypic distributions of the three SNVs from the Hardy–Weinberg equilibrium. In the three SNVs, a minor allele of each variant was associated with the reduced expression of *SLC23A2* or *SLC2A1* (Fig 2). Using the dominant model, we defined homozygote carriers of the major allele as the high expression group, and minor allele carriers as the low expression group in the subsequent analyses.

To examine the impacts of three functional variants on the risk of developing APOE4-associated cognitive decline (MCI or dementia), we assessed the APOE4-associated risk by stratification based on the genotype of each of the three variants (S4 Table), although power calculations showed limited statistical power (ranging from 32% to 55%) in each stratum. In the stratified genetic association analysis by SNV genotype (Table 2), APOE4-associated risk of developing cognitive decline was significant in the low expression group of rs1279683 (AG+AA) (*p* = 0.035, OR = 2.02, 95% CI, 1.05–3.87), whereas APOE4-associated risk was not significant in the high expression group (GG) (*p* = 0.71, OR = 1.21, 95% CI, 0.44–3.37). In contrast, both high expression groups of *SLC2A1* (rs710218; TT, rs841851; AA) showed a significant APOE4-associated risk of developing cognitive decline (rs710218: *p* = 0.037, OR = 2.35, 95% CI, 1.05–5.23, rs841851: *p* = 0.0012, OR = 3.2, 95% CI, 1.58–6.46), whereas none of the low expression groups (rs710218: TA+AA, rs841851: AG+GG) showed a significant APOE4-associated risk of developing cognitive decline (rs710218: *p* = 0.49, OR = 1.3 and 95% CI, 0.62–2.75, rs841851: *p* = 0.39, OR = 0.67, 95% CI, 0.27–1.67) (Table 2).

**Table 2:** Odds ratios of APOE4 for developing MCI and dementia, stratified by genotype of VC transporter genes.

Gene symbol	SNP ID	Genotype group	APOE4		
			Odds ratio	95% CI	*p* value
*SLC2A1*	rs710218	TT	2.35	1.05–5.23	0.037 *
		TA+AA	1.3	0.62–2.75	0.49
	rs841851	AA	3.2	1.58–6.46	0.0012 *
		AG+GG	0.67	0.27–1.67	0.39
*SLC23A2*	rs1279683	GG	1.21	0.44–3.37	0.71
		GA+AA	2.02	1.05–3.87	0.035 *

Odds ratio of APOE4 was calculated by multivariate logistic regression analysis, adjusted for effects of baseline age, follow-up period, baseline MMSE points, and sex.

* *p* < 0.05.

Table 3 shows the results of genetic association analysis of three functional variants of *SLC2A1* and *SLC23A2* with cognitive decline (MCI or dementia) in the dominant model and log-additive model. In the multivariate logistic regression analysis, none of the SNVs were significantly associated with cognitive decline in either models.

**Table 3:** Results of association analysis of three functional variants of *SLC2A1* and *SLC23A2* with cognitive decline (MCI or dementia).

Gene symbol	SNP ID	Reference	Effect	Odds ratio	95% CI	*p* value
Dominant model						
*SLC2A1*	rs710218	TT	AA+TA	1.51	0.95–2.38	0.081
	rs841851	AA	GG+AG	1.09	0.68–1.76	0.72
*SLC23A2*	rs1279683	GG	AA+GA	0.93	0.58–1.51	0.77
Log-additive model						
*SLC2A1*	rs710218	T	A	1.15	0.82–1.62	0.43
	rs841851	A	G	1.02	0.68–1.51	0.94
*SLC23A2*	rs1279683	G	A	1.13	0.82–1.58	0.45

Odds ratio of the functional variant was calculated by multivariate logistic regression analysis, adjusted for the effects of APOE4 phenotype, baseline age, follow-up period, baseline MMSE points, and sex. The upper half shows the results of the dominant model and the lower half shows the results of the log-additive model.

## Discussion

This case–control study involved the Japanese cohort population to determine the association between VC transporter genes (*SLC23A2*, *SLC2A1*, and *SLC2A3*) and APOE4-associated risk of developing cognitive decline (MCI or dementia). For this purpose, we found three functional SNVs associated with the changes in *SLC2A1* and *SLC23A2* expression by searching several publicly available databases, and analyzed the effects of these variants on APOE4-associated risk of developing cognitive decline. For the first time, we found subgroups in which APOE4 was not a significant risk factor for cognitive decline, stratified by the SNV genotype, although the statistical power was limited to detect the APOE4-associated risk in each stratified analysis. On the contrary, no association was found between functional SNVs and the risk of developing cognitive decline.

We hypothesized that genetic variants that alter the function of VC transporters in the brain also affect the VC level in the brain and APOE4-associated risk of developing cognitive decline. We applied strict criteria to identify gene variants that could affect gene functions and expression in the available databases. Similar to our study, methods for finding variants associated with the regulation of target gene expression have been reported using various databases [35]. We identified three functional SNVs in *SLC23A2* and *SLC2A1*, with abundant evidence of eQTLs for the target genes. However, we could not find any functional SNVs of *SLC2A3* using the present strict criteria. In addition, we found no large-scale SVs, SNVs, and small indels altering protein sequences in the target genes using 15% frequency threshold. The results of this database search do not imply that variants that alter the function and expression of *SLC2A3* in humans are absent. Variant filtering based on population frequency was important for our cohort size, and future studies should examine low-frequency functional variants of the brain-expressed VC transporters in larger populations with well-powered replication strategies.

The effects of APOE4 on cognitive decline (MCI or dementia) were clearly different among subgroups stratified by genotypes rs1279683, rs710218, and rs841851. The results showed that the functional variants of *SLC2A1* and *SLC23A2* may affect or modify the risk of developing APOE4-associated cognitive decline. As for *SLC23A2*, in the stratified analysis by genotype, APOE4 had a significant risk of cognitive decline in the low expression group of rs1279683, but not in the high expression group. A previous study has shown that in transgenic mice expressing additional copies of SVCT2 (encoded by *SLC23A2*), the expression of *SVCT2* mRNA and the VC levels in organs including the brain were increased accordingly, up to two-fold in the brain depending on the mRNA expression [36]. In addition, heterozygous knockout mice of SVCT2 (encoded by *SLC23A2*) showed VC deficiency in the brain [37], suggesting that the VC level in the brain changes according to *SLC23A2* expression. Cognitive function was reduced in *SVCT2* heterozygous knockout mice, and further reduced in APP_SWE_/PSEN1_ΔE9_ mice (animal AD model) crossed with *SVCT2* heterozygous knockout mice [37]. These findings suggest that the VC level in the brain and cognitive function can be dependent on *SLC23A2* expression, and cognitive function can be affected by the brain VC level, especially in animal models with a genetic risk of AD. Whether the rs1279683 genotype in *SLC23A2* changes the VC level in the human brain is unclear, and differences in brain *SLC23A2* expression might be the reason for susceptibility to cognitive decline in this study.

Contrary to *SLC23A2*, in the stratified analysis by genotype in *SLC2A1*, APOE4 was shown to have a significant risk of cognitive decline in the high expression groups of *SLC2A1* (rs710218 and rs841851), but not in the low expression groups. SLC2A1 (also called GLUT1) is expressed in the blood–brain barrier (BBB) and transports DHA bidirectionally [38]; however, whether the DHA level in the brain increases or decreases depending on the amount of this transporter, is unclear. The effects of DHA on the brain tissue have not been fully elucidated. In addition, as SLC2A1 is a non-specific DHA transporter and transports not only DHA but also sugars [23], the present results might not be because of the DHA transport function of SLC2A1. To the best of our knowledge, no study has shown that *SLC2A1* knockout in animal models alters the *in vivo* concentration and transport efficiency of DHA or ASC. Therefore, the effect of high expression of *SLC2A1* on the amount of ASC or DHA in the brain tissues remains unknown. As the first step in interpreting these results, careful investigation of the relationship among SLC2A1, DHA, and cognitive function using genetically modified animal models is necessary.

Previous studies have reported various mechanisms underlying the effects of APOE4 on AD and other dementing disorders; APOE4 affects (1) Aβ aggregation and clearance, (2) tau phosphorylation and tangle formation, (3) lipid/cholesterol transport and clearance, (4) glucose and mitochondrial metabolism, (5) inflammation, (6) vascular function including BBB, (7) insulin and VEGF signaling, and (8) synaptic and neuronal function [39]. VC has been reported to affect cognitive function in animal models and humans. In a mouse model of AD and VC deficiency in the brain (transgenic APP/PSEN1 mice with heterozygous *SVCT2* knockout), VC deficiency exacerbated cognitive performance and Aβ deposition [37]. In addition, higher VC could prevent Aβ deposition, oligomerization, BBB disruption, and mitochondrial oxidant stress in an AD mouse model [13, 14, 40, 41]. These data suggest that VC protects normal cognitive function, particularly in *APOE ɛ4*-positive individuals with Aβ deposition and oligomerization, BBB disruption, and mitochondrial oxidant stress. In humans, several prospective cohort studies have shown a significant association between higher blood VC level or antioxidant supplements, including VC, and less cognitive decline over time [42–49]. Another study reported that VC supplements in combination with NSAIDs reduced the cognitive decline in APOE4 carriers [49]. However, no randomized controlled studies have revealed the beneficial effects of VC supplementation on cognitive function [50, 51].

This study has some limitations. First, the number of participants was small as we set a high MAF (>15%) for the variants analyzed, which prevented us from testing the interaction effects in the multivariate analysis. Second, the cohort was single without replication. The third limitation is a low follow-up rate. Compared with the follow-up cases, lost cases were older, had lower MMSE scores, and had a shorter education period. The high nonparticipation and low follow-up rate suggest the potential for non-responder bias. Fourth, we did not identify the cause of dementia and MCI such as AD, dementia with Lewy bodies, and vascular dementia. Fifth, we did not include questionnaires on dietary nutrient intake, including VC. Sixth, we could not analyze data stratified by sex because of the small number of participants in this study. Additional large-scale studies in well-powered replication cohorts involving neuroimaging, neuropathology, and questions about dietary nutrients are required to understand the underlying mechanisms of the effects of functional SNVs of VC transporter genes on APOE4-associated risk of developing AD. In addition, as factors controlling the VC level in the body are thought to be complex, future analyses should take into account various factors including other genetic factors such as *SLC23A1*, which has been reported to be associated with blood VC levels in humans. These further studies will provide a deeper understanding of the relationship between VC and cognitive function in humans.

In conclusion, APOE4 significantly affects cognitive decline (MCI or dementia) in the Japanese population, and the effects are significantly different between subgroups stratified by genotype for each of the three SNVs (rs1279683, rs710218, and rs841851), which were eQTLs for the brain-expressed VC transporters (SLC23A2 or SLC2A1). Our results imply that the functional SNVs of VC transporters can affect APOE4-associated risk of developing cognitive decline via altered VC levels in the brain.

## Supporting information

S1 Table Odds ratios for developing MCI or dementia (2014–2016) in cognitively normal participants at baseline (2006–2008). (DOCX)

S2 Table Characteristics at baseline survey of the participants of the follow-up survey and subjects lost to follow-up. (DOCX)

S3 Table Genotype frequencies of *SLC2A1* and *SLC23A2* functional variants between the cognitive decline and normal cognition groups. (DOCX)

S4 Table Frequency of APOE E4-positive individuals in each stratum based on genotype of each of the three functional variants of VC transporters. (DOCX)
