# Supplementary material for: Effects of functional variants of vitamin C transporter genes on apolipoprotein E E4-associated risk of cognitive decline: The Nakajima study
Source: PLoS One. 2021 Nov 15;16(11):e0259663. doi: 10.1371/journal.pone.0259663 (PMC8592483; doi:10.1371/journal.pone.0259663)
Supplement: S1 Table — (DOCX) [file pone.0259663.s001.docx]

**S1 Table. Odds ratios for developing MCI or dementia (2014-2016) in cognitively normal participants at baseline (2006–2008).**

|  | Odds ratio | 95% CI | *p* value |
| --- | --- | --- | --- |
| APOE E4 positive (Male & Female) | 1.91 | 1.10–3.33 | 0.027 * |
| Baseline age (yrs) | 1.11 | 1.06–1.16 | <0.001 * |
| Follow-up period (yrs) | 1.26 | 1.00–1.57 | 0.046 * |
| Education period (yrs) | 0.94 | 0.84–1.06 | 0.334 |
| Baseline MMSE (points) | 0.80 | 0.71–0.90 | <0.001 * |
| Hypertension | 0.66 | 0.41–1.07 | 0.097 |
| Hyperlipidemia | 0.73 | 0.43–1.24 | 0.25 |
| Baseline Vitamin C (Standardized) | 0.81 | 0.63–1.03 | 0.087 |
| Sex | 1.59 | 0.97–2.63 | 0.068 |

Multivariate logistic regression analysis was performed to assess the independent association between APOE E4 phenotype and cognitive decline with adjustments for other variables with univariate *p* < 0.1 in Table 1. * *p* < 0.05.
