# Supplementary material for: Effects of functional variants of vitamin C transporter genes on apolipoprotein E E4-associated risk of cognitive decline: The Nakajima study
Source: PLoS One. 2021 Nov 15;16(11):e0259663. doi: 10.1371/journal.pone.0259663 (PMC8592483; doi:10.1371/journal.pone.0259663)
Supplement: S2 Table — (DOCX) [file pone.0259663.s002.docx]

**S2 Table. Characteristics at baseline survey of the participants of the follow-up survey and subjects lost to follow-up.**

|  | | Success to follow-up | Lost to follow-up | *p* value |
| --- | --- | --- | --- | --- |
| N (Male/Female) | | 400 (132/268) | 330 (129/201) | 0.089 |
| Baseline age | | 72.7 ± 5.3 | 75.2 ± 5.9 | 4.17 × 10^-9^ * |
| Baseline Vitamin C (µg/mL) | |  |  |  |
|  | Male | 5.27 ± 3.06 | 5.47 ± 3.08 | 0.60 |
|  | Female | 8.05 ± 3.31 | 8.61 ± 3.89 | 0.11 |
| Education period (yrs) | | 9.68 ± 2.26 | 9.03 ± 2.27 | 0.0043 * |
| Baseline MMSE (points) | | 27.4 ± 2.1 | 26.6 ± 2.3 | 6.21 × 10^-7^ * |
| APOE E4 positive, N(%) | | 84 (21.1%) | 59 (18.1%) | 0.35 |

Statistical analysis was performed by chi-square test and Student’s *t*-test between the groups. * *p* < 0.05.
