# Supplementary material for: Effects of functional variants of vitamin C transporter genes on apolipoprotein E E4-associated risk of cognitive decline: The Nakajima study
Source: PLoS One. 2021 Nov 15;16(11):e0259663. doi: 10.1371/journal.pone.0259663 (PMC8592483; doi:10.1371/journal.pone.0259663)
Supplement: S3 Table — (DOCX) [file pone.0259663.s003.docx]

**S3 Table.** **Genotype frequencies of *SLC2A1* and S*LC23A2* functional variants between the cognitive decline and normal cognition groups.**

| Gene symbol | SNP ID | Genotype | Cognitive decline | | Normal cognition | | HWE *p* value |
| --- | --- | --- | --- | --- | --- | --- | --- |
|  |  |  | n = 141 | | n = 247 | |  |
|  |  |  | Genotype count | (Frequency) | Genotype count | (Frequency) |  |
| *SLC2A1* | rs710218 | AA | 10 | (7.1) | 28 | (11.3) | 0.78 |
|  |  | TA | 70 | (49.6) | 88 | (35.6) |  |
|  |  | TT | 61 | (43.2) | 131 | (53.0) |  |
|  | rs841851 | GG | 5 | (3.5) | 11 | (4.5) | 0.82 |
|  |  | AG | 50 | (35.5) | 72 | (29.1) |  |
|  |  | AA | 86 | (61.0) | 164 | (66.4) |  |
| *SLC23A2* | rs1279683 | AA | 28 | (19.9) | 38 | (15.4) | 0.71 |
|  |  | GA | 64 | (45.4) | 127 | (51.4) |  |
|  |  | GG | 49 | (34.8) | 82 | (33.2) |  |

HWE, Hardy-Weinberg equilibrium.
