# Supplementary material for: Effects of functional variants of vitamin C transporter genes on apolipoprotein E E4-associated risk of cognitive decline: The Nakajima study
Source: PLoS One. 2021 Nov 15;16(11):e0259663. doi: 10.1371/journal.pone.0259663 (PMC8592483; doi:10.1371/journal.pone.0259663)
Supplement: S4 Table — (DOCX) [file pone.0259663.s004.docx]

**S4 Table.** **Frequency of APOE E4-positive individuals in each stratum based on genotype of each of the three functional variants of VC transporters.**

| Gene symbol | SNP ID | Genotype group | Normal cognition | | Cognitive decline | |
| --- | --- | --- | --- | --- | --- | --- |
|  |  |  | Total, N | APOE E4 positive, N(%) | Total, N | APOE E4 positive, N(%) |
| *SLC2A1* | rs710218 | TT | 131 | 21 (16.0%) | 61 | 18 (29.5%) |
|  |  | TA+AA | 116 | 22 (19.0%) | 80 | 22 (27.5%) |
|  | rs841851 | AA | 164 | 21 (12.8%) | 86 | 27 (31.4%) |
|  |  | AG+GG | 83 | 22 (26.5%) | 55 | 13 (23.6%) |
| *SLC23A2* | rs1279683 | GG | 82 | 13 (15.9%) | 49 | 10 (20.4%) |
|  |  | GA+AA | 165 | 30 (18.2%) | 92 | 30 (32.6%) |
